# A HER2 Tri-Specific NK Cell Engager Mediates Efficient Targeting of Human Ovarian Cancer

**DOI:** 10.3390/cancers13163994

**Published:** 2021-08-08

**Authors:** Daniel A. Vallera, Felix Oh, Behiye Kodal, Peter Hinderlie, Melissa A. Geller, Jeffrey S. Miller, Martin Felices

**Affiliations:** 1 Masonic Cancer Center, University of Minnesota, Minneapolis, MN 55455, USA; ohxxx408@umn.edu (F.O.); KODA0020@umn.edu (B.K.); phoward@umn.edu (P.H.); gelle005@umn.edu (M.A.G.); mille011@umn.edu (J.S.M.); 2 Department of Radiation Oncology, University of Minnesota, Minneapolis, MN 55455, USA; 3 Division of Hematology, Oncology, and Transplantation, Department of Medicine, University of Minnesota, Minneapolis, MN 55455, USA; 4 Division of Gynecologic Oncology, Department of Obstetrics, Gynecology and Women’s Health, University of Minnesota, Minneapolis, MN 55455, USA

**Keywords:** NK cells, HER2, ADCC, IL-15, bispecific antibodies, ovarian cancer, carcinoma, innate immunotherapy, TriKEs

## Abstract

**Simple Summary:**

HER2 is a marker known to be over-expressed on breast cancer, rendering it one of the most useful solid tumor targets for antibody-based therapies. Despite expression on ovarian cancer, results targeting HER2 in this setting have been disappointing, thus requiring more aggressive approaches. Natural killer (NK) cells are known as principal mediators of cancer cell killing, but cancer cells find ways to deter them. We devised a tri-specific biological drug containing antibody fragments that simultaneously binds NK cells and cancer cells and at the same time delivers a natural cytokine signal that triggers robust NK cell expansion. In vitro studies show the drug augments NK cell killing of a number of HER2-positive human cell lines, while enhancing NK cell activation and proliferation. Studies in mice engrafted with human ovarian cancer showed the drug has anti-tumor efficacy, clearly demonstrating its ability to bolster NK cells in their ability to contain tumor cell growth.

**Abstract:**

Clinical studies validated antibodies directed against HER2, trastuzumab, and pertuzumab, as useful methodology to target breast cancer cases where HER2 is expressed. The hope was that HER2 targeting using these antibodies in ovarian cancer patients would prove useful as well, but clinical studies have shown lackluster results in this setting, indicating a need for a more comprehensive approach. Immunotherapy approaches stimulating the innate immune system show great promise, although enhancing natural killer (NK) function is not an established mainstream immunotherapy. This study focused on a new nanobody platform technology in which the bispecific antibody was altered to incorporate a cytokine. Herein we describe bioengineered CAM1615HER2 consisting of a camelid VHH antibody fragment recognizing CD16 and a single chain variable fragment (scFv) recognizing HER2 cross-linked by the human interleukin-15 (IL-15) cytokine. This tri-specific killer engager (TriKE^TM^) showed in vitro prowess in its ability to kill ovarian cancer human cell lines. In addition, we demonstrated its efficacy in inducing potent anti-cancer effects in an in vivo xenograft model of human ovarian cancer engrafting both cancer cells and human NK cells. While previous approaches with trastuzumab and pertuzumab faltered in ovarian cancer, the hope is incorporating targeting and cytokine priming within the same molecule will enhance efficacy in this setting.

## 1. Introduction

Ovarian cancer is the most lethal gynecologic cancer in women, leading to an estimated 21,410 women being diagnosed and 13,770 women dying of the disease in 2021 [1]. Roughly three-quarters of ovarian cancer cases are diagnosed in advanced stages because it frequently affects the abdominal cavity and presents with a paucity of symptoms [2]. Despite new efforts focusing on ovarian cancer, overall survival has not improved over the past few decades [3]. Thus, new approaches are urgently needed. 

A potential target for ovarian cancer is the marker human epidermal growth factor receptor-2 (HER2), a member of the epidermal growth factor receptor (EGFR) family of transmembrane receptor tyrosine kinases. HER2 has a direct association with cancer since its overexpression is associated with poor prognosis in breast cancer and it triggers intracellular signaling pathways related to cell proliferation, differentiation, and survival [4]. In a 2018 report on ovarian cancer, 34 studies that included 5180 ovarian cancer patients were analyzed. Expression of HER2 was negatively correlated with clinical prognosis of overall survival and disease-free survival/progression-free survival [5]. In epithelial ovarian cancer, HER2 expression is more commonly found in older patients, the serous subtype, and patients with advanced stage and high-grade differentiation [6]. Clearly, HER2 is worthy of consideration as a valid ovarian cancer target. 

There are currently two HER2-targeting antibodies being used clinically: trastuzumab, which was first to be tested clinically, and pertuzumab, which is a humanized anti-HER2 antibody. These antibodies demonstrated success, alone and in combination, in breast cancer therapy, achieving in some trials an overall response rate in excess of 80% when combined with chemotherapy [7,8]. In the ovarian cancer setting these antibodies mediate antibody-dependent cellular cytotoxicity (ADCC) [9]. However, their role in HER2+ ovarian cancer has been controversial, with some studies showing modest responses, which are typical of second-round therapies in ovarian cancer. While these responses have been tepid, the agent presented here targets NK cells to HER2-expressing tumors and shows distinct advantages with its expansion abilities, thus perhaps proving useful in this arena. 

While therapeutic antibodies have been around for some time, cancer immunotherapy has recently made remarkable advances with the discovery of immune system engagers such as bi-specific T cell engagers (BiTEs), tri-specific killer engagers (TriKEs^TM^), and checkpoint inhibitors. Our contribution to cancer immunotherapy was the discovery of a methodology for expanding the bispecific antibody platform by crosslinking an anti-CD16 single chain variable fragment (scFv) and an scFv recognizing cancer antigens with the cytokine interleukin 15 (IL-15) [10]. CD16 is known to be one of the strongest activating NK cell receptors, capable of inducing activation without need for a co-stimulatory activating signal, unlike other NK cell receptors, while IL-15 has been shown to be critical in NK cell expansion, priming, and survival. Thus, TriKEs potently stimulate NK cell activation, expansion, and antigen-specific tumor killing. The first TriKE that we reported recognized CD33 expressed on liquid tumors (myeloid leukemia cells) and is currently being evaluated in the clinic in a Phase I/II trial (NCT03214666). Our group has recently improved this platform [11] and demonstrated that it can be leveraged for solid tumor targeting as well [12].

This study describes a novel HER2-targeting TriKE aimed at improving control of ovarian cancer. The data presented indicate that it potently enhances NK cell mediated control of HER2-expressing ovarian cancer in vitro and successfully inhibits tumor growth in a murine xenograft model that accommodates the engraftment of human ovarian cancer cells and human NK cells. These data support the further consideration of this CAM1615HER2 TriKE for clinical translation in the ovarian cancer setting.

## 2. Materials and Methods

### 2.1. Construction of CAM1615HER2 TriKE

Single variable domain nanobody fragments (VHH) of camelid-specific heavy chain origin offer advantages over conventional scFv fragments, including fewer disulfide linkages, higher affinity, and more compact size. Therefore, we spliced DNA fragments encoding the CDR regions from a camelid anti-CD16 [13] into a universal, humanized nanobody scaffold previously shown to allow grafts of antigen-binding loops with transfer of the antigen specificity and affinity [14]. This new sequence was used to manufacture CAM1615HER2. The fully assembled hybrid gene CAM1615HER2 (from 5′ end to 3′ end) encoded a NcoI restriction site; an ATG start codon; anti-human CD16 VHH; a 20 amino acid (aa) segment, PSGQAGAAASESLFVSNHAY; human IL-15; the seven amino acid linker, EASGGPE; anti-HER2 scFv [15], and a XhoI restriction site. The resulting hybrid gene was spliced into the pET28c expression vector under the control of an isopropyl-D-thiogalactopyranoside (IPTG) inducible T7 promoter. The DNA target gene encoding CAM1615HER2 was 1512 base pairs due to the smaller size of the camelized VHH gene. Wild-type human IL-15 was used and not a mutated form of the cytokine. The Biomedical Genomics Center, University of Minnesota, St. Paul, MN, verified the gene sequence and in-frame accuracy of the target gene. To closely evaluate the issue of specificity, we generated a Control TriKE containing the CAM1615 portions and a non-binding scFv that does not direct function against SKOV3 cells (or display degranulation and inflammatory activity against other cell lines) in the same manner as the CAM1615HER2 TriKE was generated.

### 2.2. Purification of Protein from Inclusion Bodies

*Escherichia coli* strain BL21 (DE3) (Novagen, Madison, WI, USA) was used for protein expression after plasmid transfection. The bacteria were cultured overnight in 800 mL Luria broth containing 50 μg/mL kanamycin. Expression was induced via the addition of IPTG (FischerBiotech, Fair Lawn, NJ) when the media reached an absorbance of 0.65 at 600 nm. Bacterial expression resulted in packaging of target protein into inclusion bodies. After expression, bacteria were harvested and then homogenized in buffer (50 mM Tris, 50 mM NaCl, and 5 mM EDTA pH 8.0), and the pellet was sonicated and centrifuged. To extract protein from the pellet, a solution of 0.3% sodium deoxycholate, 5% Triton X-100, 10% glycerin, 50 mmol/L Tris, 50 mmol/L NaCl, and 5 mmol/L EDTA (pH 8.0) was used, and the extract was washed 3 times.

Protein from inclusion bodies requires refolding. Thus, a sodium N-lauroyl-sarcosine (SLS) air oxidation method modified from a previously reported procedure was used [16]. Briefly, inclusion bodies were dissolved in 100 mM Tris, 2.5% SLS (Sigma, St. Louis, MO, USA). Pellets were removed by centrifugation. A total of 50 μM of CuSO_4_ was added to the solution and then incubated at room temperature with rapid stirring for 20 h for air-oxidization of –SH groups. Removal of SLS was performed by adding 6 M urea and 10% AG 1-X8 resin (200–400 mesh, chloride form) (Bio-Rad Laboratories, Hercules, CA, USA) to the detergent-solubilized protein solution. The next step added 13.3 M of guanidine HCl into protein solution and 2~3 h incubation at 37 °C. The solution was diluted 20-fold with refolding buffer, 50 mM Tris, 0.5 M l-arginine, 1 M urea, 20% glycerol, 5 mM EDTA, pH 8.0. The mixture was incubated at 4 °C for 2 days. To remove the buffer, we dialyzed against 5 volumes of 20 mM Tris-HCl at pH 8.0 for 48 h at 4 °C, then 8 volumes for 18 additional hours. Product was then purified first by fast flow Q ion exchange chromatography and then by passage over a size exclusion column (Superdex 200, GE). Final protein was stored in PBS and frozen in −80 °C. Sodium dodecyl sulfate polyacrylamide gel electrophoresis (SDS-PAGE) was performed using Simply Blue Safe Stain (Invitrogen, Carlsbad, CA, USA) to evaluate protein size and purity. Final yield was 3 mg TriKE/L of flask culture.

### 2.3. Cancer Cell Lines

The following cell lines were obtained from the American Type Culture Collection: MCF-7L (ductal breast carcinoma), MCF-7L-TamR (tamoxiphen resistant subline of MCF-7), SKOV3 (ovarian ascites), SK-BR-3 (breast carcinoma derived from metastatic site), UMSCC-11B squamous cell carcinoma derived from larynx tumor [17], and MA-148 (ovarian carcinoma). For in vivo experiments, SKOV3-luc was made by transfecting SKOV3 with a luciferase reporter construct using Invitrogen’s Lipofectamine Reagent and selective pressure applied with 10 μg/mL of blastocidin. MA148 (established locally at the University of Minnesota) is a human epithelial ovarian carcinoma cell line. Lines were maintained in RPMI 1640 RPMI supplemented with 10–20% fetal bovine serum (FBS) and 2 mmol/L L-glutamine. Lines were incubated in a humidified atmosphere containing 5% CO_2_ at a constant 37 °C. When the adherent cells were more than 90% confluent, they were passaged using trypsin-EDTA for detachment. For cell counts, a standard hemocytometer was used. Only those cells with a viability > 95% were used for the experiments, as determined by trypan blue exclusion.

### 2.4. Cell Products

Peripheral blood mononuclear cells (PBMCs) were obtained from normal volunteers, while ascites cells were obtained from patients after consent was received, and institutional review board (IRB) approval was granted (protocols 9709M00134 and STUDY00011437), in compliance with guidelines by the Committee on the Use of Human Subjects in Research and in accordance with the Declaration of Helsinki. For xenogeneic mouse studies, fresh PBMCs were magnetically depleted three times (i.e., three pass-throughs across the magnet) of CD3 and CD19-positive cells, according to the manufacturer’s recommendations (STEMCELL Technologies, Cambridge, MA, USA), to generate an NK-cell-enriched product. Ovarian cancer specimens (ascites) were collected from women diagnosed with advanced-stage ovarian or primary peritoneal carcinoma at time of primary debulking surgery. Cells were pelleted, lysed for red blood cells, cryopreserved in 10% DMSO/90% FBS, and stored in liquid nitrogen.

### 2.5. Evaluation of NK Cell Activation and Tumor Cytotoxicity

NK cell activation against tumor targets in the presence or absence of treatments was measured in a flow cytometry assay by evaluating degranulation via CD107a (lysosomal-associated membrane protein LAMP-1) and intracellular interferon-gamma (IFNγ) production [18,19]. Upon thawing, normal donor PBMCs or patient-derived ascites cells were rested overnight (37 °C, 5% CO_2_) in RPMI 1640 media supplemented with 10% fetal calf serum (RPMI-10). The next morning, they were suspended with tumor-target cells or media after washing twice with RPMI-10. Cells were then incubated with TriKEs or controls for 10 min at 37 °C. Fluorescein isothiocyate (FITC)-conjugated anti-human CD107a monoclonal antibody (BD Biosciences, San Jose, CA, USA) was then added. Following an hour of 37 °C incubation, GolgiStop (1:1500, BD Biosciences) and GolgiPlug (1:1000, BD Biosciences) were added for 4 h. After washing with phosphate-buffered saline, the cells were stained with PE/Cy 7–conjugated anti-CD56 mAb, APC/Cy 7–conjugated anti-CD16 mAb, and PE-CF594–conjugated anti-CD3 mAb (BioLegend, San Diego, CA, USA). Cells were incubated for 15 min at 4 °C, washed, and fixed with 2% paraformaldehyde. Cells were then permeabilized using an intracellular perm buffer (BioLegend) to evaluate production of intracellular IFNγ through detection via aBV650 conjugated anti-human IFNγ antibody (BioLegend). Samples were washed and evaluated in an LSRII flow cytometer (BD Biosciences, San Jose, CA, USA). Flow cytometric analysis was carried out on FlowJo v10 (FlowJo LLC., Ashland, OR, USA). NK cells in all flow cytometry studies were gated with the following strategy: singlets/LiveDead-/FSC-SSC Lymph gate/CD56^+^CD3^−^.

### 2.6. NK Cell Expansion via IL-15 Stimulation 

To measure the potency of the IL-15 moiety within the TriKE, PBMCs from healthy donors were labeled with CellTrace Violet Proliferation Dye (Invitrogen, Carlsbad, CA, USA) according to kit specifications. After staining, effector cells were cultured with 50 nM of TriKEs or controls and incubated in a humidified atmosphere containing 5% CO_2_ at 37 °C for 7 days. Cells were harvested, stained for viability with Live/Dead reagent (Invitrogen, Carlsbad, CA, USA), and surface stained for anti-CD56 PE/Cy7 (Biolegend, San Diego, CA, USA) and anti-CD3 PE-CF594 (BD Biosciences, Franklin Lakes, NJ, USA) to gate on the viable CD3^−^CD56^+^ NK cell population and CD3^+^CD56^−^ T cell population. Data analysis was performed using FlowJo software (Flowjo Enterprise LCC, version 7.6.5, Ashland, OR, USA).

### 2.7. Spheroid Assays

Tumor killing was evaluated in real-time using the IncuCyte S3 platform. A total of 20,000 SKOV3-GFP cells/well were plated in 96-well round-bottom ultra-low adhesion (ULA) plates (Corning, Flintshire, UK) and allowed to form spheroids for 3 days. A total of 40,000 magnetic-bead-enriched NK cells were then added to each well. Noted treatments were then added at a 30 nM concentration, and the plate was placed in an IncuCyte S3^®^ platform housed inside a cell incubator at 37 °C/5% CO_2_. Images from three technical replicates were taken every 45 min for 72 h using a 4× objective lens and then analyzed using IncuCyte™ Basic Software v2018A (Sartorious). Graphed readouts represented target spheroid size and intensity, normalized to live spheroids alone at the starting (0 h) time point.

### 2.8. In Vivo Mouse Study and Imaging

The efficacy of the CAM1615HER2 TriKE was tested in a xenogeneic mouse model that was previously reported [10], but it was modified for the growth of the human ovarian cancer cell line SKOV3. The line was transfected with a luciferase reporter gene to permit monitoring of tumor progression via bioluminescent imaging in real time. NSG mice (NOD.Cg-Prkdc^scid^ Il2rg^tm1Wjl^/SzJ, *n* = 5/group) were injected IP with 2.0 × 10^5^ SKOV3-luc tumor cells and then 3–5 days later received low-dose total body irradiation (275 cGy). The following day, all groups received enriched NK cells (PBMC magnetically CD3 and CD19 depleted) and began drug treatment. A single course of treatment consisted of 50 μg of drug given IP 5×/week (MTWThF) for two weeks and then maintenance therapy 3×/week (MWF) through day 60. Mice were imaged weekly. At each imaging session, mice were injected with 100 μL of 30 mg/mL luciferin substrate 10 minutes before and then imaged under isoflurane gas sedation. Imaging data were gathered using Xenogen Ivis 100 imaging system with Living Image 2.5 analysis software (Xenogen Corporation, Hopkington, MA, USA).

### 2.9. Statistical Analysis

GraphPad PRISM (GraphPad Prism Software, Inc., La Jolla, CA, USA) was used to create all statistical tests. Unless otherwise noted, all multiple comparison studies used one-way ANOVA with repeated measures, while single comparison studies used Student *t* test. Error bars display mean ± SEM, and statistical significance was shown as * *p* < 0.05, ** *p* < 0.01, *** *p* < 0.001, and **** *p* < 0.0001.

## 3. Results

### 3.1. Generation of CAM1615HER2 TriKE

**Figure 1:**
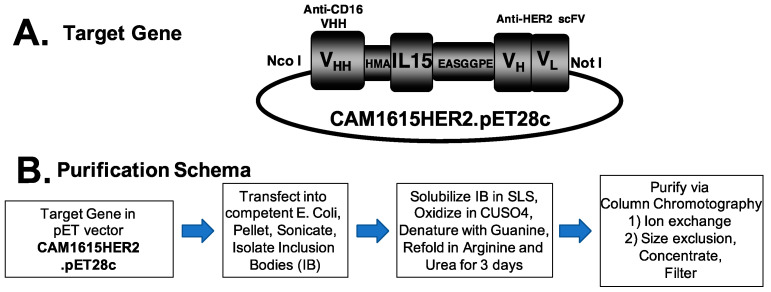
Methodology for generation of CAM1615HER2 TriKE. (**A**) Schematic depicting CAM1615HER2 consisting of (left to right) camelid anti-CD16 VHH, human IL-15, and anti-HER2 scFv. (**B**) Flow chart of TriKE production and purification schema.

TriKEs are composed of three separate binding regions including a single domain antibody (VHH) that binds CD16, an scFv that binds a tumor antigen present on cancer cells, and cross-linked wildtype human IL-15 that mediates cytokine signaling on the NK cells. Using this scaffold, a TriKE targeting HER2, called CAM1615HER2, was generated using a pET expression vector (Figure 1A). Once the vector was assembled, it was expressed in *E. coli*, inclusion bodies (IB) were isolated and solubilized, and TriKE protein was then refolded and purified using Ion exchange and size exclusion chromatography (Figure 1B). Supplementary Figure S1A shows the absorbance tracing of the fractions of bacterial and target protein as it passes over FFQ ion exchange column as the first phase of purification from inclusion bodies. Eluant was collected in 8 mL aliquots, shown on the abscissa of the graph. The double-sided arrow shows the target peak collected as the drug exited the column. Supplementary Figure S1B shows the absorbance tracing from the second purification phase, size exclusion chromatography (SEC). Again, the double-sided arrow marks the peak collected as final product. The final product (fractions C2-D4) formed a mostly pure (90%) single band when analyzed using SDS-PAGE with Coomassie Blue staining, providing evidence of a uniform product with a molecular weight of about 55 kDa (Supplementary Figure S1C).

### 3.2. Evaluation of Proliferation Mediated by CAM1615HER2 TriKE

**Figure 2:**
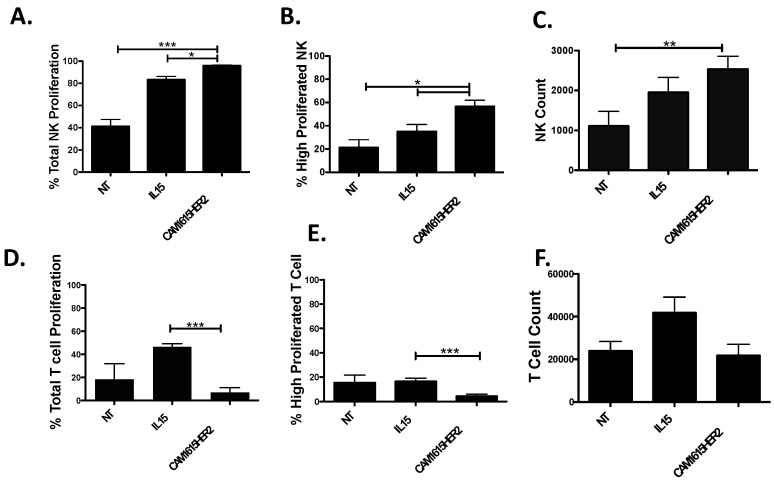
CAM1615HER2 TriKE induces specific NK cell expansion. PBMCs were CellTrace labeled and put in culture with nothing (NT), recombinant human IL-15 (IL15; 30 nM), or the CAM1615HER2 TriKE (30 nM) for 7 days (*n* = 6). Cells were then harvested and stained for surface markers to differentiate proliferation on NK cells (CD3^−^CD56^+^; **A**–**C**) or T cells (CD3^+^CD56^−^; **D**,**E**). The first column of panels shows the total proportion of cells that proliferated (**A**,**D**), the second column shows the proportion of cells that proliferated beyond three divisions (**B**,**E**), and the final column shows the total number of cells present (**C**,**F**). Error bars display mean ± SEM, and statistical significance was shown as * *p* < 0.05, ** *p* < 0.01, and *** *p* < 0.001.

The human IL-15 moiety within the TriKE is meant to specifically mediate IL-15 signaling on NK cells; thus, the ability of the CAM1615HER2 TriKE to drive NK expansion was determined. PBMCs were labeled with CellTrace dye and placed in culture with noted treatments for 7 days to determine how the TriKE influences NK cell versus T cell proliferation, measured by dilution of the dye. When compared to the no treatment control or treatment with rhIL-15, the CAM1615HER2 TriKE enhanced overall proliferation (Figure 2A), proliferation of NK cells past three rounds of division (Figure 2B), and resulted in the highest number of NK cells at the end of the assay (Figure 2C). Conversely, when evaluating T cells, unlike the rhIL-15 treatment, the CAM1615HER2 TriKE treatment did not expand T cells (Figure 2D,E). Together, these studies indicated that the CAM1615HER2 TriKE specifically stimulates expansion of NK cells, and not T cells, and that IL-15 within the TriKE was functional and in a viable conformational alignment. 

### 3.3. Determination of CAM1615HER2 TriKE Specificity and Function

**Figure 3:**
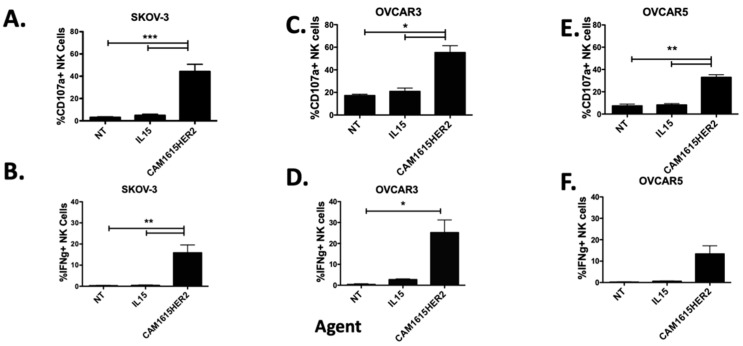
CAM1615HER2 TriKE mediates NK cell activation against ovarian cancer cell lines. To determine the ability of the CAM1615HER2 TriKE to induce NK cell activation against ovarian cancer cells, PBMCs were incubated with SKOV3 cells (**A**,**B**), OVCAR3 cells (**C**,**D**), and OVCAR5 cells (**E**,**F**) for 5 h with noted treatments (30 nM) and degranulation (CD107a: **A**,**C**,**E**), and intracellular cytokine production (IFNγ: **B**,**D**,**F**) was evaluated on NK cells by flow cytometry (*n* = 4). NT = No treatment (NK alone) and IL15 = treatment with recombinant IL-15. Error bars display mean ± SEM, and statistical significance was shown as * *p* < 0.05, ** *p* < 0.01, and *** *p* < 0.001.

TriKE molecules mediate immunotherapy via formation of a cytolytic bridge that triggers ADCC on the NK cell against a tumor cell expressing the TriKE targeted antigen, HER2 in this instance. To establish TriKE specific activity against HER2 expressing cells, NK cell activation was measured by evaluation of NK cell degranulation (CD107a) and inflammatory cytokine production (IFNγ) when PBMCs were incubated with tumor HER2+/− targets and the CAM1615HER2 TriKE or controls. When incubated with CAM1615HER2 TriKE, NK cells produced substantially more CD107a and IFNγ against HER2-positive SKOV3 and SK-BR-3 cells when compared to controls (Figure 3A,B and Supplementary Figure S2A,B). Conversely, no differences in NK cell degranulation or inflammatory cytokine production were seen between the CAM1615HER2 TriKE and controls when the PBMCs were incubated with the HER2-negative line UM-SCC-11B cell line (Supplementary Figure S2C,D). Of note, pre-incubation of NK cells and SKOV3 targets with a high concentration of HER2 scFv preceding TriKE treatment robustly impacted CAM1615HER2 TriKE NK cell activation, as noted by a 59% decrease in degranulation when taking into account background of natural cytotoxicity (Supplementary Figure S2E). To compare the HER2-targeting standard of care, Herceptin, to the CAM1615HER2 TriKE, both were included in assays against breast cancer (SK-BR-3) and ovarian cancer (SKOV-3) cells, and both drugs induced comparable NK cell activation (Supplementary Figure S2A,B and Figure 2F,G respectively). When compared to controls, the CAM1615HER2 TriKE also showed increased CD107a and IFNγ NK cell activity against a broad panel of ovarian cancer cell lines including OVCAR3 (Figure 3C,D), OVCAR5 (Figure 3E,F), OVCAR8 (Supplementary Figure S3A,B), and OVCAR4 (Supplementary Figure S3C,D) cell lines. The aforementioned assays were carried out at saturating concentrations of TriKE (30 nM), perhaps masking differences in activity mediated by ligand expression on the ovarian cancer cell targets. To determine if HER2 antigen expression levels impact NK cell activation via the TriKE, a limiting dose of CAM1615HER2 (0.3 nM) was added to the assay and tested against a high density HER2 ovarian cancer cell (SKOV-3) versus low-density lines (OVCAR3 and OVCAR5). Results indicated that antigen density impacted NK cell activation via CAM1615HER2 as the TriKE induced more CD107a and IFNγ on NK cells against SKOV-3 cells than OVCAR3 or OVCAR5 cells (Supplementary Figure S3E,F). The CAM1615HER2 TriKE also demonstrated activity against a broad panel of HER2-expressing tumors including pancreatic (BxPC-3), prostate (C4-2), lung (NCI-H322), and breast (MCF-7L) cancer (Supplementary Figure S4). Taken together, these data demonstrated broad applicability of the CAM1615HER2 TriKE against a variety HER2-expressing tumors.

### 3.4. CAM1615HER2 TriKE Induces Dynamic Evaluation of Ovarian Cancer Spheroids 

**Figure 4:**
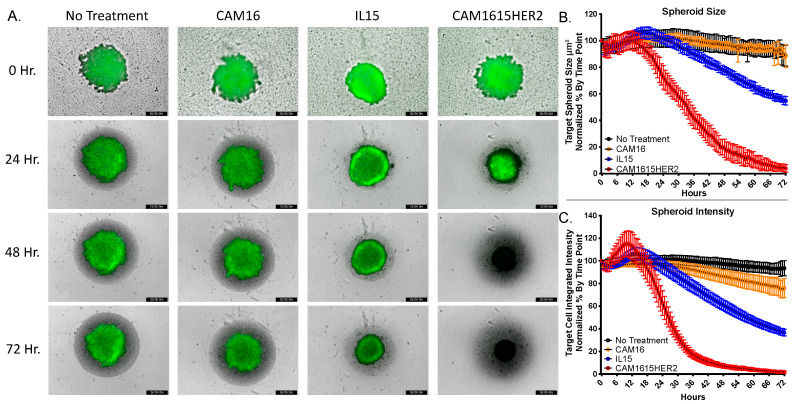
CAM1615HER2 TriKE induces enhanced ovarian cancer spheroid killing. GFP-expressing SKOV3 cells were plated in 96-well ULA plates and allowed to form spheroids for 3 days within the IncuCyte S3 at 37 °C/5% CO_2_. Magnetically enriched NK cells were added in the presence of no treatment, 30 nM CAM16 VHH, 30 nM IL-15, or 30 nM CAM1615HER2 TriKE. Spheroids were then imaged every 45 min over a three-day period. (**A**) Representative spheroid images with noted treatments at 0, 24, 48, and 72 h post treatment. (**B**) Quantification of spheroid size and (**C**) intensity over a 72 h period normalized by % change (of tumor alone) at each time point (two separate experiments with 7 technical replicates per condition per experiment).

To determine the ability of the CAM1615HER2 TriKE to amplify NK cell mediated killing of ovarian tumors, the IncuCyte S3 platform was employed. This automated imaging platform allows for dynamic evaluation of spheroid killing, via quantitative measurement of spheroid size and intensity, over an extended period of time. GFP-expressing SKOV3 cells were plated in ultra-low adhesion (ULA) plates and allowed to form for three days. Enriched NK cells were then added in the presence of no treatment, a CAM16 single domain antibody, recombinant human IL-15, or the CAM1615HER2 TriKE. Spheroid size and intensity was then tracked for a period of three days. The CAM1615HER2 TriKE induced dynamic and robust killing of the tumor, noted by changing of the spheroid color from green (live SKOV3 cells) to black (dead SKOV3 cells), within 48 h, while the IL-15 group induced less killing over the three-day period but more than the no treatment and CAM16 controls (Figure 4A). Quantification of spheroid size and fluorescent intensity further highlights the differences in tumor killing mediated by NK cells treated with the CAM1615HER2 TriKE versus cells treated with the no treatment, CAM16, or IL15 controls (Figure 4B,C). Taken together, these data showed the potency of the CAM1615HER2 TriKE treatment against multicellular tumor formations.

### 3.5. CAM1615HER2 TriKE Amplifies Function of NK Cells Derived from the Tumor Microenvironment

**Figure 5:**
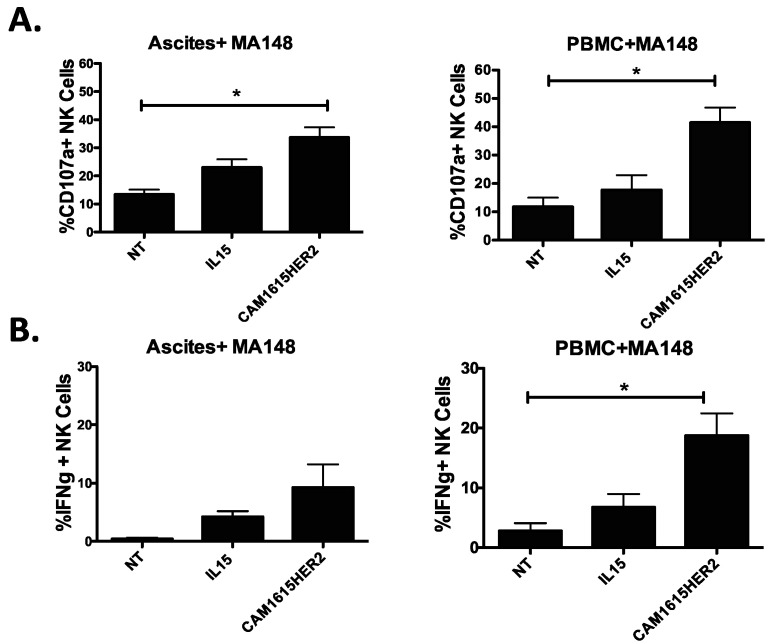
CAM1615HER2 TriKE can activate ovarian cancer patient NK cells. To evaluate the ability of the CAM1615HER2 TriKE to induce activity on NK cells from the relevant tumor microenvironment, ascites (left column) was obtained from high-grade ovarian cancer patients at the time of surgical debulking and compared to PBMCs (right column) from normal donors. Cells were incubated with MA148 ovarian cancer cells and noted treatments (at 30 nM) for 5 h and surface CD107a expression (**A**) and intracellular IFNγ expression (**B**) were evaluated on NK cells by flow cytometry (*n* = 9). Error bars display mean ± SEM, and statistical significance was shown as * *p* < 0.05.

Our group has previously shown that NK cells are present in relatively similar proportions with several similar characteristics in the ovarian cancer microenvironment, yet their activity is strongly impacted in that setting [20]. To evaluate if the CAM1615HER2 TriKE can rescue NK cell anti-tumor activity from ovarian cancer patients, we obtained high-grade ovarian cancer patient ascites cells at the time of cytoreductive surgery and incubated them with TriKE or controls and MA148 cells, a high-grade serous ovarian carcinoma cell line established at the University of Minnesota [21]. Compared to no treatment and IL-15 controls, the CAM1615HER TriKE statistically induced more degranulation (CD107a) on ascites NK cells against MA148 targets (Figure 5A, left). It is important to note however that rescue was not complete when comparing to the level of degranulation seen on normal donor PBMC NK cells treated with the CAM1615HER2 TriKE, highlighting the lasting negative of effects of the ovarian cancer tumor microenvironment on NK cell function (Figure 5A, right). Similarly, the CAM1615HER2 TriKE amplified ascites NK cell inflammatory cytokine production (IFNγ) against MA148s more than controls, but not to the level seen with normal donor PBMC NK cells (Figure 5B). Thus, although the ovarian cancer tumor microenvironment limits maximal NK cell function, in part due to decreased CD16 NK expression in that environment [20], the CAM1615HER2 TriKE still amplified function on these cells, thus demonstrating clinical relevance. 

### 3.6. CAM1615HER2 TriKE Demonstrates Tumor Control in Xenogeneic Ovarian Cancer Model

**Figure 6:**
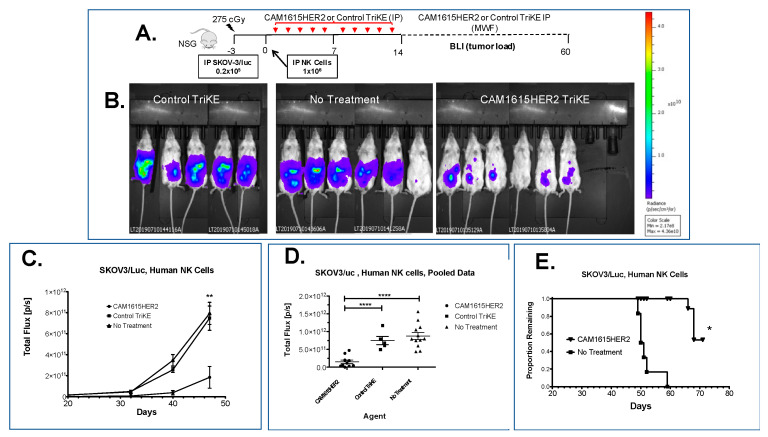
CAM1615HER2 TriKE mediates ovarian cancer control in the xenogeneic model. (**A**) Schematic of SKOV3-luc xenogeneic NSG mouse model with enriched NK cells. (**B**) Representative BLI images of different treatment groups (control TriKE, CAM1615HER2 TriKE, and no treatment) at about day 40 after the beginning of treatment. (**C**) Single experiment line graph of BLI images throughout the study until day 47, where the last BLI was taken (*n* = 5–7 mice/group). (**D**) Scatter plot of pooled BLI data combining two experiments showing tumor load at day 47 of mice treated with CAM1615HER2 TriKE (*n* = 10), control TriKE (*n* = 5), or no treatment (*n* = 12) when HER2 TriKE treatment is compared to control TriKE treatment or no treatment controls. (**E**) Kaplan Meier survival curve of confirmatory experiment demonstrating survival of animals within each treatment group (*n* = 6). Error bars display mean ± SEM, and statistical significance was shown as * *p* < 0.05, ** *p* < 0.01, and **** *p* < 0.0001.

The in vivo efficacy of the CAM1615HER2 TriKE was determined in xenogeneic mouse models containing human NK cells and SKOV3 cells (Figure 6A). Briefly, NSG mice were irradiated and engrafted intraperitoneally (IP) with luciferase-expressing SKOV3 tumor cells prior to receiving a dose of enriched human NK cells (also IP). Mice were then treated with nothing (no treatment), a control TriKE that did not induce activation against SKOV-3 but did induce proliferation (Supplementary Figure S5A–C), or CAM1615HER2 TriKE. Evaluation of tumor load at about day 40 using bioluminescent imaging (BLI) showed a clear differential between the mice that received CAM1615HER2 TriKE and the ones that received only NK cells or NK cells plus the control TriKE, demonstrating higher BLIs in the latter groups (Figure 6B). BLI tracking over the course of the experiment demonstrated that tumor load between the three groups started segregating around day 30, with maximal differences noted at about day 47, with the CAM1615HER2 TriKE demonstrating highly significant and superior tumor control (Figure 6C,D). It is important to note that while the control TriKE is capable of inducing NK cell expansion, it demonstrated reduced tumor control when compared to the CAM1615HER2 TriKE. Survival tracking after the end of BLI showed that five of six animals in the untreated group died by day 52, while there was still a 50% survivorship by day 72 in the CAM1615HER2 TriKE treated group (Figure 6E). The difference was significant by the Student *t* test (*p* < 0.05). Together, these data confirmed that the CAM1615HER2 TriKE is efficacious in inhibiting the growth of human ovarian carcinomas in a xenogeneic model and that the in vivo efficacy was highly specific.

## 4. Discussion

Surgical and chemotherapeutic approaches to the treatment of distal ovarian cancer remain largely ineffective, demanding new approaches to this lethal gynecologic malignancy [22]. Immunotherapeutic approaches that offer specific targeting of the cells to the tumor, via exploitation of tumor antigens, are a promising path to resolving this issue. One such tumor antigen is HER2. In ovarian cancer, levels of HER2 expression are controversial, but ovarian cancer is a complex disease with many histological subtypes including serous, mucinous, endometrioid, and clear cell cancer. HER2 expression can differ according to subtype. For example, HER2 positivity is higher in serous (29%) and mucinous carcinoma (38%) compared to endometrioid (20%) and clear cell carcinoma (23.1%) [23]. Thus, reported differences may be an issue of cancer subtype. Still, the association between HER2 expression and poor ovarian cancer prognosis is well recognized [24,25,26]. 

HER2 (Epidermal Growth Factor Receptor 2, HER2/neu, CD340) targeting via trastuzumab (Herceptin) or pertuzumab has demonstrated relatively good levels of success in breast cancer [5]. Additionally, it has been used to treat patients presenting with HER2+ gastric cancer [27] and esophageal cancer (ClinicalTrials.gov Identifier: NCT02954536). Trastuzumab, the first agent targeting HER2, was first approved for medical use in the United States over twenty years ago and since has been included on the World Health Organization’s List of Essential Medicines comprising the most effective and safe medicines needed in healthcare [28]. Early clinical studies of trastuzumab showed significant overall survival in late-stage (metastatic) HER2-positive breast cancer from 20.3 to 25.1 months [29], and regarding early stage HER2-positive breast cancer, it reduced the risk of relapse following surgery. Clearly, trastuzumab has shown immense value in treatment of HER2-positive metastatic breast cancer [30,31]. Adverse events are remarkably low.

Utilization of trastuzumab and pertuzumab in ovarian cancer has resulted in far less impressive clinical outcomes, despite confirmed HER2 expression in many of these settings [32]. Thus, we set out to alter the mechanism of action for targeting the tumor in this setting. While antibodies can mediate NK ADCC, natural killer cells differ from T cells in that activating receptor triggering alone, in this case through CD16, does not induce NK cells expansion and survival [9]. Higher NK cell numbers within the ascites of ovarian cancer patients have been correlated to increased survival and better outcomes [33]; therefore, we hypothesized that combining an HER2-targeted ADCC with an NK specific proliferation signal would greatly enhance endogenous NK cell activity in the ovarian cancer setting. Thus, there is a good rationale for combining HER2 targeting therapeutic antibodies with IL-15. However, while recombinant human IL-15 and IL-15 receptor complexes are actively being evaluated in the clinic, untargeted delivery of IL-15 has been shown to have some toxicities and induce robust expansion of CD8 T cells that would compete for IL-15 with the NK cells meant to mediate ADCC [34,35,36,37,38]. A molecule that drives targeted delivery of IL-15 to NK cells and mediates HER2 targeting could circumvent this issue. The CAM1615HER2 TriKE molecule described here does just that. Our pre-clinical data show that the CAM1615HER2 TriKE induces activity against HER2-expressing tumors, activates NK cells via CD16 resulting in specific killing of ovarian cancer spheroids, drives specific NK cell expansion, and induces control of the ovarian tumor in vivo. Although no overt toxicity was noted with the TriKE treatment group, as assessed by enhanced survival in this group, it is important to note that the TriKE does not engage mouse CD16 or HER2. The SKOV3luc xenogeneic model used has some other limitations, including likely lack of focalized tumor at the time of NK cell injection and start of treatment. However, it is clear the CAM1615HER2 TriKE was efficient at controlling the tumor and enhancing survival in this setting. Future studies, utilizing better established focalized tumor models, might better shed light onto how well the TriKE can function in the solid tumor microenvironment.

Of particular relevance to immunotherapeutic approaches in this setting, the ovarian cancer tumor microenvironment is suppressive and can affect the functional characteristics of local immune cells [39]. This includes NK cells, and we and others have shown a decrease in CD16 expression on NK cells derived from the ascites of ovarian cancer patients [20,39,40]. CD16 mediates ADCC on NK cells, so it is plausible, at least in the context of the patient NK cells, that this phenomenon is responsible for failure of trastuzumab and pertuzumab to drive tumor clearance in ovarian cancer patients. This is also the likely reason why we saw reduced activity with the CAM1615HER2 TriKE on ascites NK cells, versus the normal donor peripheral blood NK cells, albeit we did see significant increases in activity when compared to the controls. While the CD16 reduction in expression within the ovarian tumor microenvironment may impact the ADCC mechanism of the TriKE, and the therapeutic antibodies, it has been shown that localization of IL-15 at the site of the tumor results in enhanced NK cell activity against the tumor and tumor clearance [41]. We and others have shown that IL-15 can enhance natural cytotoxicity on NK cells [20,33,42,43], a process that is independent of CD16 expression, so inclusion of the IL-15 moiety within the CAM1615HER2 TriKE not only induces NK cell expansion but also provides NK cells priming for a killing mechanism alternative to ADCC. While other therapeutic approaches are being taken to target HER2, such as utilization of CAR T cells [44,45,46], it is important to consider that HER2 expression can also be found in normal ovarian cancer cells [47], raising concerns with safety. However, NK cells have specifically evolved to avoid killing of normal cells through inhibitory receptors on NK cells that interact with cognate HLA on normal cells, adding an extra level of safety to NK cell targeted approaches [7,8]. Thus, toxicities should not be worse than those seen with trastuzumab and pertuzumab. Taken together, these findings argue that the CAM1615HER2 TriKE displays unique characteristics capable of improving on the HER2-targeting antibodies currently in the clinic. Currently, a Phase I/II clinical trial testing a first-generation TriKE, targeting CD33 in myeloid malignancy settings, is underway, and we hope to obtain safety data on this platform soon (CinicalTrials.gov NCT03214666). Our data argue that the CAM1615HER2 TriKE should be tested clinically next in ovarian cancer and other solid tumor settings where HER2 overexpression is present (breast, lung, etc.) with the hope of providing an alternative approach to reduce mortality in these setting.

## 5. Conclusions

In conclusion, this study focuses on a unique platform technology, incorporating IL-15 as a bispecific antibody cross-linker, thus driving NK-cell-mediated targeting of cancer. TriKEs overcome non-specific mechanisms of natural NK cell cytotoxicity by promoting an antigen-specific synapse resulting in enhanced NK cell-mediated killing, activation, and expansion. HER2 has proven to be one the most useful biological drug targets benefiting thousands of breast cancer patients and was the focus of this study. In both in vitro and in vivo studies, our second-generation HER2 TriKE demonstrated impressive potency and significant anti-tumor effects. The data were particularly important regarding our favorable findings in ovarian cancer models. Previous testing of trastuzumab and pertuzumab faltered in ovarian cancer, but there is possibility that targeting, priming, and precipitous expansion of NK cells promoted by the same molecule will enhance efficacy, even against this difficult solid tumors. Thus, an important contribution of this work is the implementation of a HER2 TriKE to target HER2-positive ovarian cancer and supports of its clinical translation. 

## Data Availability

Data is contained within the article or supplementary material.

## Supplementary Materials

The following are available online at https://www.mdpi.com/article/10.3390/cancers13163994/s1, Figure S1: Determination of CAM1615HER2 TriKE Specificity and Function. Figure S2: Determination of CAM1615HER2 TriKE Function against additional HER2+ ovarian cancer cell lines. Figure S3: Determination of CAM1615HER2 TriKE Function against additional HER2+ cancer cell lines. Figure S4: Activity of Negative Control TriKE. Sequences Patent application WO2021055342A1.
