# Supplementary material for: A HER2 Tri-Specific NK Cell Engager Mediates Efficient Targeting of Human Ovarian Cancer
Source: Cancers (Basel). 2021 Aug 8;13(16):3994. doi: 10.3390/cancers13163994 (PMC8394622; doi:10.3390/cancers13163994)
Supplement: Supplementary file 1 [file cancers-13-03994-s001.zip › cancers-1255886_supplementary.pdf]

cancers

MDPI

Supplemental Materials

A HER2 tri-specific NK Cell Engager mediates efficient targeting of human ovarian cancer

Daniel A. Vallera [1,2,*], Felix Oh [1,2], Behiye Kodal [1,3], Peter Hinderlie [1,3], Melissa Geller [1,4], Jeffrey S. Miller [1,3], and Martin Felices [1,3,*]

1 Masonic Cancer Center, University of Minnesota, Minneapolis, MN 55455
2 Department of Radiation Oncology, University of Minnesota, Minneapolis, MN 55455; ohxxx408@umn.edu (F.O.); (D.A.V.)
3 Department of Medicine, Division of Hematology, Oncology, and Transplantation, University of Minnesota, Minneapolis, MN 55455; KODA0020@umn.edu (B.K.); phoward@umn.edu (P.H.); mille011@umn.edu (J.S.M.); (M.F.)
4 Department of Obstetrics, Gynecology and Women's Health, Division of Gynecologic Oncology, University of Minnesota, Minneapolis, MN 55455; gelle005@umn.edu (M.A.G.)
* Correspondence: valle001@umn.edu (D.A.V.); mfelices@umn.edu (M.F.)

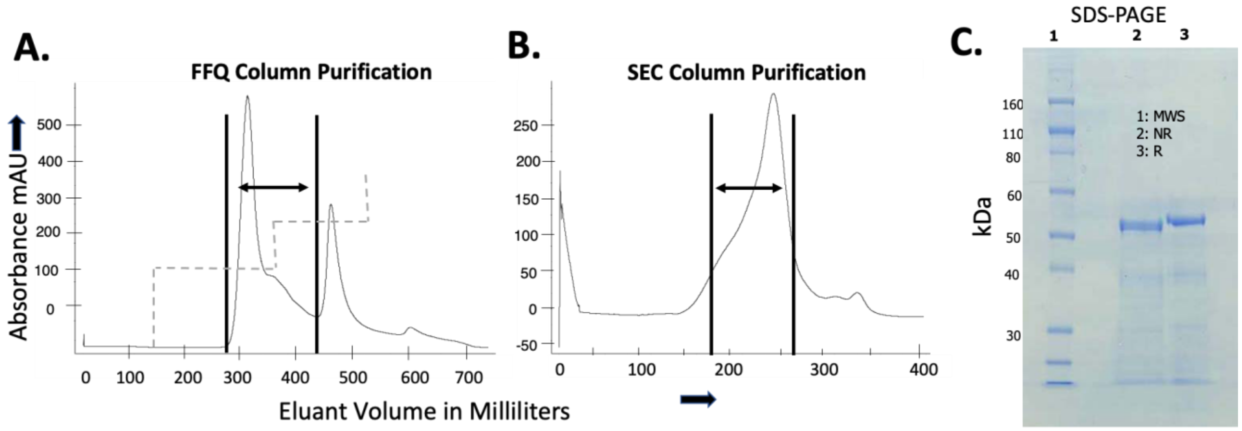

Figure S1. Evaluation of CAM1615HER2 purification. A. Chromatography trace resulting from the first-step purification of CAM1615HER2 on an ion exchange (FFQ) column. The collection peak is indicated by the double-sided arrow. B. Chromatography trace of resulting from the second-step purification of CAM1615HER2 on size exclusion column. The collection peak is indicated by the double-sided arrow. C. SDS-PAGE gel stained with Coomassie Blue Dye indicating the purity and size of the final product after the two orthogonal column steps. MWS – Molecular Weight Standards, NR – nonreduced, R – Reduced.

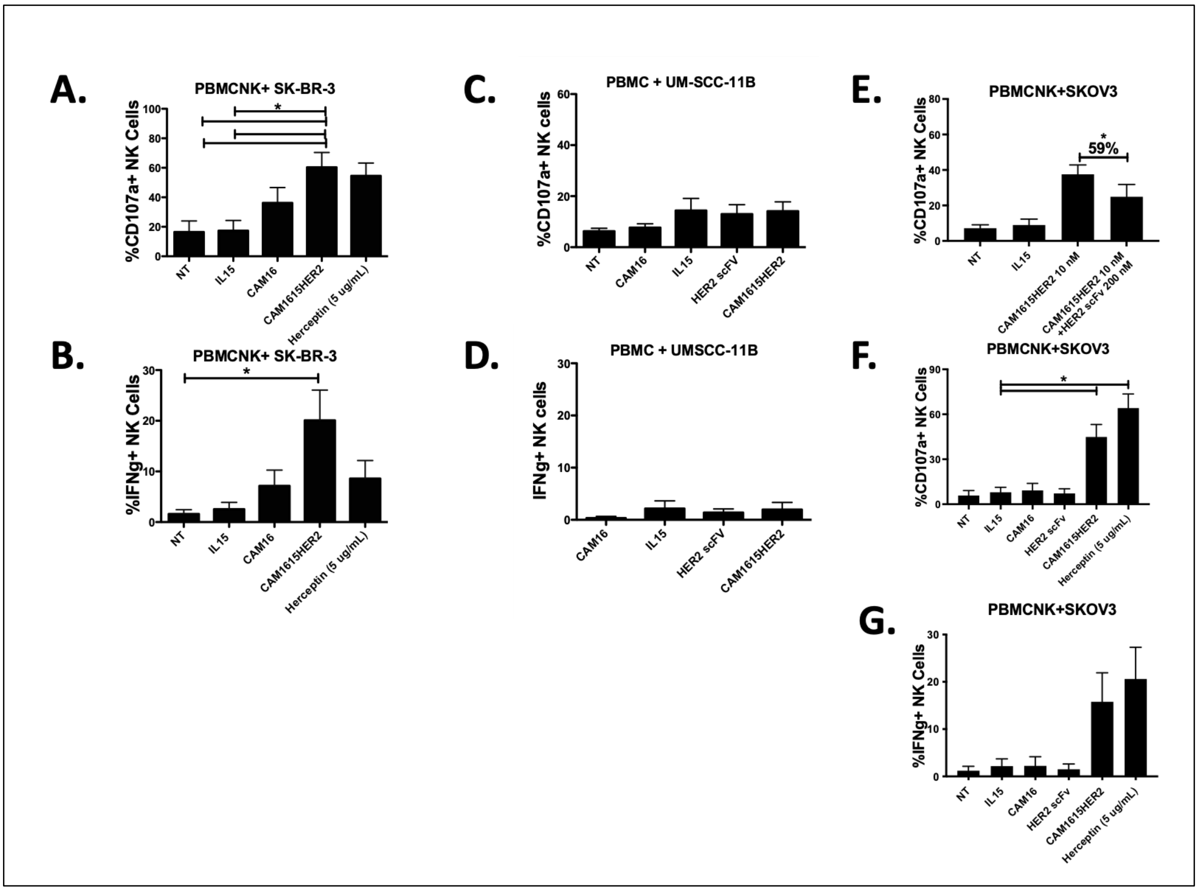

Figure S2. Determination of CAM1615HER2 TriKE Specific Function. To determine the ability of the CAM1615HER2 TriKE, or noted controls, to induce NK cell activation PBMCs were incubated with HER2 positive SK-BR-3 cells for 5 hours and degranulation (CD107a: A.) and intracellular cytokine production (IFNγ: B.) were evaluated with treatments at 30 nM concentration unless otherwise noted. To determine the ability of the CAM1615HER2 TriKE, or noted controls, to induce NK cell activation PBMCs were incubated with HER2 negative UM-SCC-11B cells for 5 hours and degranulation (CD107a: C.) and intracellular cytokine production (IFNγ: D.) were evaluated with treatments at 30 nM concentration unless otherwise noted. E. To evaluate specificity of binding against a HER2 positive Ovarian cancer cell line in a functional assay PBMCs were incubated with SKOV3 cells in the presence or absence of 200 nM anti-HER2 scFv for 10 minutes prior to addition of 10 nM CAM1615HER2 TriKE. After a 5 hour co-culture degranulation on the NK cells was assessed by flow cytometry. Percent blockade of function by the HER2 scFv was calculated by the following formula: % Blocking = (1-((block-NT)/(not blocked-NT))*100%. F. and G. To determine the ability of the CAM1615HER2 TriKE, or noted controls, to induce NK cell activation, PBMCs were incubated with SKOV3 cells for 5 hours and degranulation (CD107a: F.) and intracellular cytokine production (IFNγ: G.) were evaluated with treatments at 30 nM concentration unless otherwise noted. (N=4). NT = No treatment (NK alone) and IL15 = treatment with recombinant IL15.

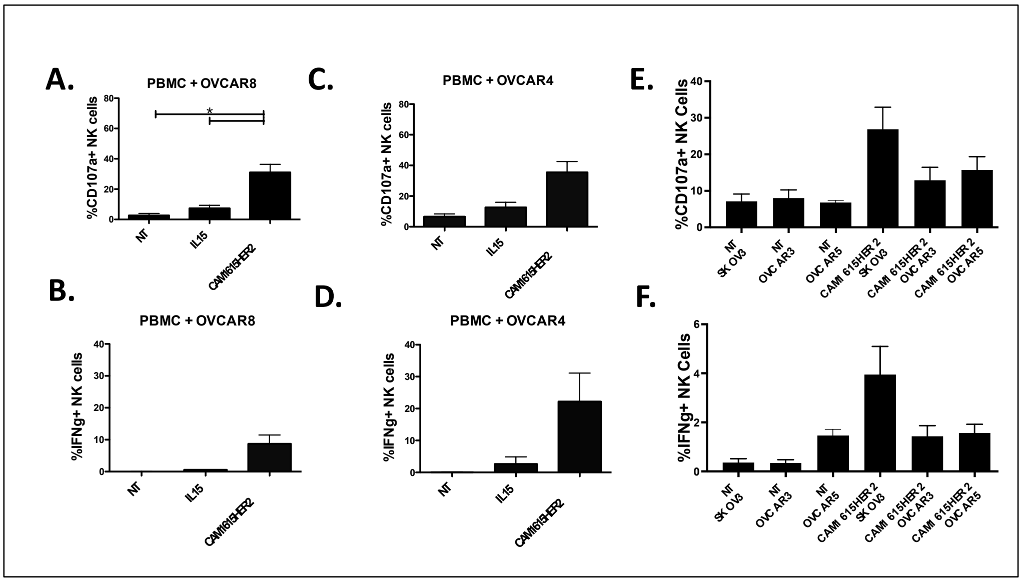

Figure S3. Determination of CAM1615HER2 TriKE Function against additional HER2+ ovarian cancer cell lines. To determine the ability of the CAM1615HER2 TriKE (30 nM), or noted controls (30 nM for IL-15), to induce NK cell activation against ovarian cancer cells, PBMCs were incubated with OVCAR8 cells (A. and B.) or OVCAR4 cells (C. and D.) for 5 hours and degranulation (CD107a: A. and C.) and intracellular cytokine production (IFNγ: B. and D.) was evaluated on NK cells by flow cytometry. E. and F. To determine the impact of tumor antigen density on TriKE mediated NK cell activation, high density (SKOV3) and low density (OVCAR3 and OVCAR5) ovarian cancer targets were incubated with limiting (0.3 nM) TriKE or no treatment and degranulation (E.) and IFNγ production (F.) was measured on NK cells after 5 hours. (N=4). NT = No treatment (NK alone) and IL15 = treatment with recombinant IL-15.

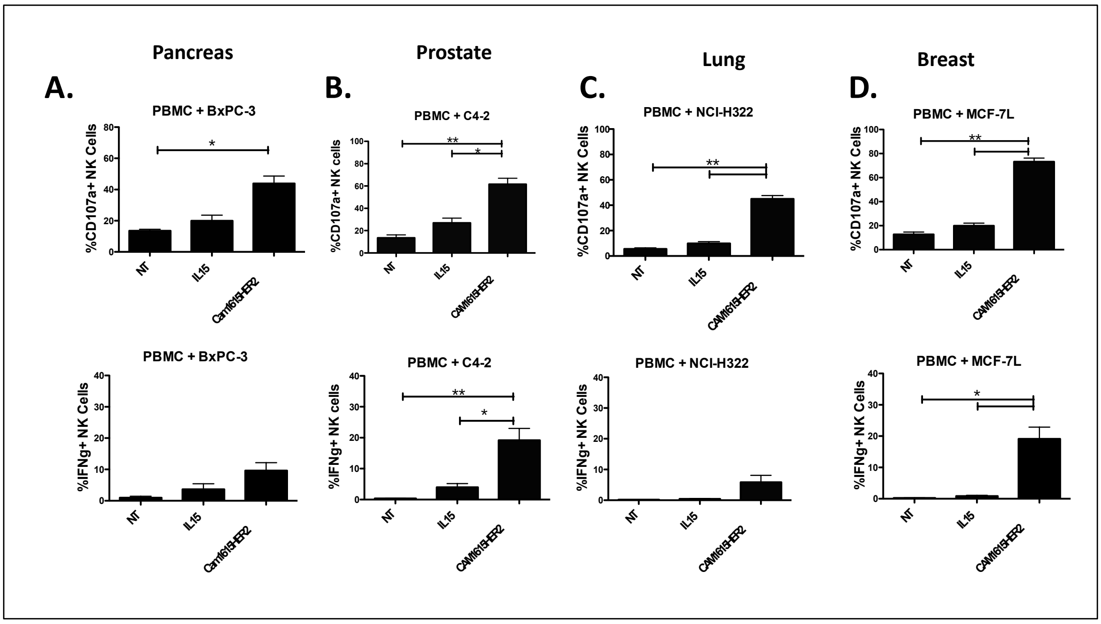

Figure S4. Determination of CAM1615HER2 TriKE Function against additional HER2+ cancer cell lines. To determine the ability of the CAM1615HER2 TriKE, or noted controls, to induce NK cell activation against a broad panel of tumors, PBMCs were incubated with BxPC-3 cells (A.), C4-2 cells (B.), NCI-H322 cells (C.), or MCF-7L cells (D.) for 5 hours and degranulation (CD107a: top panels) and intracellular cytokine production (IFNg: bottom panels) was evaluated on NK cells by flow cytometry (N=4). NT = No treatment (NK alone) and IL15 = treatment with recombinant IL-15.

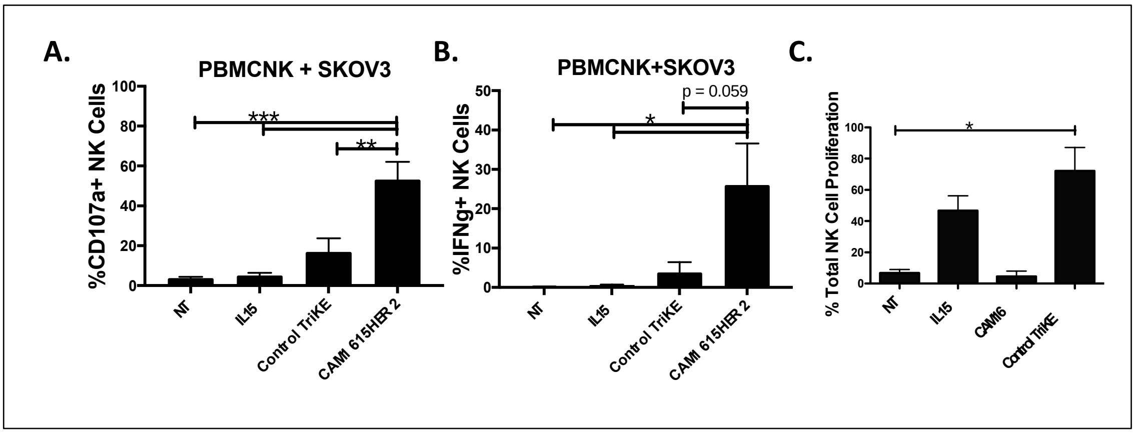

Figure S5. Activity of Negative Control TriKE. To determine the activity of the negative Control TriKE used in xenograft experiments to kill ovarian cancer functional assays were carried out against SKOV3 cells, (A) CD107a and (B) IFNγ, and proliferation was evaluated separately over a 7 day period by evaluating CellTrace dilution (C). in (A) CD107a assay, (B) IFNγ assay, and (C) a proliferation assays were carried out. The Control Trike was prepared identically to the HER2 TriKE with a non-reactive antibody (scFv) fragment. The Control TriKE was not active against SKOV3. *p<0.004 when TriKE is compared to the NT Group or the IL15-treated group.
